# Time to subsequent therapy (TTST) as an endpoint in clinical studies: development of standardized documentation of subsequent therapy through systematic literature review, expert interviews, and Delphi survey

**DOI:** 10.1007/s00432-026-06583-w

**Published:** 2026-09-02

**Authors:** Bianca Biedenweg, Thorsten Ruppert, Thomas Kohlmann, Martin Danner, Anja Mehnert-Theuerkauf, Diana Lüftner, Ulrike Filippig, Janine Ziemann, Ulrike Osowski, Peter Kaskel

**Affiliations:** 1 Dept. Methods in Community Medicine, Institute for Community Medicine, University Medicine Greifswald, Greifswald, Germany; 2 German Association of Research-Based Pharmaceutical Companies (vfa), Berlin, Germany; 3 BAG SELBSTHILFE German Federal Working Group for Self-Help for People with Disabilities, Chronic Illnesses and Their Relatives (Registered Association Under German Law), Düsseldorf, Germany; 4 Dept. of Med. Psychology and Med. Sociology, University Medical Center Leipzig, Leipzig, Germany; 5 Immanuel Klinik Märkische Schweiz, Buckow, Germany & Immanuel Klinik Rüdersdorf, Medical University of Brandenburg, Rüdersdorf bei Berlin, Germany; 6 Onkolotsin, Fehmarn, Germany; 7 Merck Healthcare Germany GmbH, Darmstadt, Germany; 8 MSD SHARP & DOHME GMBH, Munich, Germany

**Keywords:** Benefit assessment, Endpoint, Oncology, Patient relevance, Time to subsequent therapy, TTST

## Abstract

**Background:**

The endpoint Time to Subsequent Therapy (TTST) is an intermediate endpoint used in research and regulatory assessments. TTST denotes initiation of subsequent therapy and is a clearly definable, clinically relevant event for healthcare professionals. However, it has not been systematically established to which extent TTST is subjectively meaningful to patients. The objective of this study was to define TTST as a patient-relevant intermediate endpoint.

**Methods:**

The study examined five oncological indications (breast cancer, prostate cancer, melanoma, multiple myeloma, and non-small cell lung cancer) using a systematic literature review, analysis of case report forms used in international randomized controlled trials, review of German Federal Joint Committee (G-BA) documents, semi-structured interviews and a two-stage Delphi survey with healthcare professionals, patients, and relatives.

**Results:**

A total of 35 individuals participated in qualitative interviews. Most of them rated TTST as particularly significant. The Delphi Survey included 264 interviewees in round one, and 117 in round two. Patient-relevance of TTST was confirmed by 81% of respondents (95% confidence interval 76%, 85%). Nine treatment scenarios that justify TTST were identified. To capture patient-relevance, prospective collection of reasons for and consequences of therapy change are required. A checklist with standardized response formats plus free-text fields was developed: a comprehensive master checklist for flexible, complete documentation and a short version focused on therapy change-specific items.

**Conclusions:**

TTST is an intermediate endpoint whose systematic documentation of characteristics demonstrating patient-relevance can be standardized in research and clinical practice using the developed checklists.

**Supplementary Information:**

The online version contains supplementary material available at https://doi.org/10.1007/s00432-026-06583-w.

## Introduction

The time to subsequent therapy (TTST) is the time from the index treatment to the initiation of subsequent therapy (Agapow et al. 2023). TTST is a clearly definable and clinically relevant event that is of high importance for both patients and healthcare professionals (Agapow et al. 2023; Branchoux et al. 2022; Campbell et al. 2020). Accordingly, Time to Subsequent Therapy (TTST) has become established as an intermediate, care-relevant endpoint in clinical research (Agapow et al. 2023; Bahlis et al. 2020; Österborg et al. 2016). TTST was specifically referenced by the German Federal Joint Committee (G-BA) in the initial assessment of olaparib (Olaparib 2015) and is also used in international health services research (Rivera et al. 2022). From a regulatory perspective, capturing subsequent therapies is essential to adequately assess the benefit-risk profile of a treatment (Merino et al. 2023; https://www.ema.europa.eu/en/documents/other/joint-htab-regulatory-perspectives-understanding-evidence-challenges-managing-uncertainties-exploring-potential-solutions_en.pdf). In the German healthcare context, an endpoint is considered patient-relevant if it is subjectively meaningful and perceptible to patients (Biomarkers Definitions Working Group 2001). TTST should be regarded as an independent, healthcare relevant intermediate endpoint.

TTST is typically defined as the interval from initiation of the index therapy to the start of the next systemic treatment. “Subsequent therapy” hereby is used for the next line of treatment and “therapy change” for the transition itself. TTST is used as a patient-centered outcome reflecting the duration of clinical benefit from therapy, but definitions and estimation methods vary across studies.

This project was designed primarily for the German HTA context. Since 2015, TTST has been reported by pharmaceutical companies in 73 German Pharmaceutical Market Reorganization Act (AMNOG) procedures (AMNOG-Monitor 2025), but the G-BA has not yet recognized the endpoint as patient-relevant because corresponding empirical evidence is lacking (Olaparib 2015). To address this evidence gap, University of Greifswald initiated a project in 2018, funded by the German Association of Research-Based Pharmaceutical Companies (vfa) and coordinated by the former vfa Working Group Endpoints in Oncology of the vfa Research and Development Committee. The aim of the project was to explore and substantiate the patient relevance of TTST and to develop a methodologically robust checklist to standardize collection and documentation of patient-relevant aspects related to therapy changes and subsequent therapies in both health-services research and randomized clinical trials (RCTs).

## Methods

The project focused on five oncological tumor types: Breast cancer, prostate cancer, melanoma, multiple myeloma, and non-small cell lung cancer. These indications were selected because substantial information was available (e.g., historical data, public dossier assessments, treatment pathways, and identified treatment gaps).

TTST is usually analyzed using standard time-to-event methods with initiation of subsequent therapy as the event of interest. Details on censoring, competing events and handling of treatment restarts should be specified in the study protocol or statistical analysis plan.

The project was conducted in Germany because TTST is particularly relevant in the German HTA context, where patient relevance and benefit assessment require explicit documentation of patient-centered aspects. At the same time, the checklist was intended as a transferable framework for international clinical studies, as the underlying questions about treatment change, patient involvement, and therapy consequences are broadly relevant across healthcare settings.

A multi-stage mixed-methods research approach was chosen to address the project objective from complementary perspectives. The systematic literature review in German and English sources, the analysis of anonymized Case Report Forms (CRFs) from international industry RCTs, and the review of G-BA benefit assessment documents identified existing evidence and contextualized TTST within regulatory, clinical and HTA practice; the semi-structured expert interviews generated in-depth qualitative insights into relevant therapy-change aspects; and the two-stage Delphi survey (Häder 2014) validated, prioritized and structured these findings, culminating in a practice-oriented checklist for general use.

The TTST endpoint was considered in relation to the clinical question addressed by the study and the corresponding estimand. Events such as disease progression, treatment discontinuation, death, or other factors influencing the start of subsequent therapy were considered conceptually when defining and documenting TTST. Accordingly, their handling should be understood as part of the endpoint’s operationalization rather than as a formal competing-risk analysis.

Statistical analysis was performed using SPSS.

## Literature review

A systematic literature review (SLR) of German and English publications (PubMed; 2009–2019; see Table A.1 [search terms] and Table A.2 [hits] in the online Appendix), using predefined search terms (see Supplementary information), was performed December 2018–March 2019. The results informed a semi-structured interview guide and a two-stage Delphi survey. To update the evidence base for publication, an additional PubMed literature search covering publications from 2019 to 2025 was conducted between March and April 2025. Consequently, the overall literature review finally covers the period from 2009 to 2025.

De-identified CRFs from international RCTs conducted with registration intent (provided by the former vfa Working Group Endpoints in Oncology) were reviewed.

The G-BA public website was systematically searched for documents on completed benefit assessments according to §35a of the Fifth Book of the German Federal Social Code (SGB V) in the oncological areas that mentioned the TTST endpoint, with attention to prior rationale for classifying TTST as not patient-relevant by G-BA (for orphan drugs) or by the Institute for Quality and Efficiency in Health Care (IQWiG; for non-orphan drugs) (see Table A.3 in the online Appendix).

### Development of expert interview guides

Semi-standardized interview guides, letters of information and consent forms were developed from the SLR. Participants were purposely sampled from all German federal states based on expertise (medicine, psycho-oncology, health economics, nursing, oncology navigation (Porzig et al. 2018), patient representatives) via systematic online searches and professional networks (https://www.oncomap.de/centers?showMap=1). Invitations were sent directly. The medical and psycho-oncological professionals had scientific expertise, worked in interdisciplinary oncology centers or certified Comprehensive Cancer Centers (CCC) in Germany (see OncoMap (Porzig et al. 2018)) and could draw on many years of experience with clinical studies. Patients and advocates were additionally recruited through self-help groups and decentralized patient advisory councils (via the Cancer Information Service (https://www.krebsinformationsdienst.de/adressen-und-links/selbsthilfegruppen).

Pre-interviewing (for quality checking) started in March 2019 with the main interview phase undertaken from August 2019 to March 2020 by two researchers (interviewer and recorder). Content analysis captured all potentially relevant topics for a documentation tool. Interview results informed items for the first Delphi round (Mayring 2019).

### Nationwide Delphi survey

The two-stage online Delphi survey (Häder 2014) (using the LimeSurvey platform) aimed to (1) identify which therapy changes are relevant to the TTST endpoint; (2) quantify its relevance; (3) rank documentation content; (4) propose operationalization options for a checklist; and (5) clarify technical documentation aspects of documentation. Recruitment mirrored the interview strategy, selecting experienced professionals nationwide. The first round was conducted August—October 2020, and the second round July—September 2021. Survey items were based on interview findings. The online tool was pre-tested for functionality.

### Development of the checklist

The checklist was developed iteratively from SLR, interviews, and Delphi results, compared with de-identified CRFs and G-BA/IQWiG requirements for TTST, and mapped to the following four therapy-change time points: (1) time before therapy change; (2) time of decision; (3) time of therapy change; and (4) time after implementation of therapy change. The vfa Endpoints in Oncology Working Group reviewed and made suggestion to revise the master checklist. A short checklist for use in international RCTs was also produced.

The study was reviewed by the Ethics Committee of University Medicine Greifswald (internal register BB 090/19) issued on July 31, 2019. All participants provided written informed consent.

## Results

### Systematic literature review and screening of documents

The SLR identified 15 relevant publications and 12 G-BA benefit assessments referencing the TTST endpoint. The 12 benefit assessments covered breast cancer (3), multiple myeloma (3), non-small cell lung cancer (4), and prostate carcinoma (2). As for the melanoma appraisals reviewed, TTST had not been included.

G-BA/IQWiG cited several reasons for not considering TTST, including: (1) missing follow-up of patient-relevant parameters (quality of life, symptoms, side effects) after therapy change; (2) delays in subsequent therapy due to side effects of the drug under review; (3) initiation of subsequent therapy based solely on imaging procedures; (4) overlap with other endpoints preventing independent evaluation; (5) inadequate documentation of treatment decisions; (6) post-hoc definition of the endpoint; (7) inclusion of adverse events (AE) from subsequent therapies; (8) failure to account for mortality or time to first chemotherapy; and (9) investigator unblinding after completion of therapy. For direct inclusion, TTST should therefore be pre-defined, distinct from other endpoints, and demonstrably patient-relevant (e.g., through documentation of patient involvement in treatment decisions).

Analysis of exemplary CRFs from regulatory RCTs showed routine standardized collection of start and end of subsequent therapy, respectively, timing and reasons for therapy change (patient-reported reasons such as side effects, or medical reasons like progression), type of change (surgery vs. new medication), setting (inpatient vs. outpatient), outcome of the change, and further subsequent measures.

### Expert interviews

Interviews were conducted with 15 medical or psycho-oncological professionals; six nursing professionals, patient navigators, or health economists; and 14 patients, relatives, or patient advocates. Face-to-face interviews (conducted by TK, BB, JZ) lasted 20–75 min (median 60’, mean 53’).

Physicians’ and psycho-oncologists’ perspectives varied by tumor type, treatment strategy, and research context. Nursing professionals and patient navigators emphasized communication gaps: Patients often felt inadequately informed and excluded from shared decision-making. Patients, relatives, and advocates consistently wished for stronger inclusion of personal preferences, fears, life plans (e.g., fertility concerns), daily life, and social context in therapy discussions.

Overall, respondents regarded TTST as an objectively meaningful and highly patient-relevant endpoint, supporting documentation of various therapy-change aspects. Table 1 summarizes documentation elements relevant for the operationalization of TTST as an intermediate, care-relevant endpoint (and not for surrogate validation).

**Table 1:** Summary of significant aspects of a therapy change from the conducted interviews

Core interview topic	Significant aspect of therapy change
Patient-specific parameters	Previous therapies, intolerances to previous therapies Co-medications Co-morbidities, pre-existing conditions Disease progression Pregnancy Previous transplantation(s) Behavioral aspects Domestic and social environment Education level Psychosocial status Health literacy
Side effects and stage of disease	Symptoms Laboratory parameters (tumor markers, organ function) Imaging results Tumor growth and invasiveness Metastases Toxicity
Therapy decision	Reasons for change Therapy perspectives (risk, benefit, prognoses, limitations, etc.) Tumor board recommendations Consensus vs. subgroup-specific vs. individual therapy recommendations Willingness of the attending physician Involvement of patients, consideration of their wishes, goals and fears Involvement of other people (type and extent) Content of informed consent discussions Questions from patients and their relatives
Subsequent therapy	Type, extent, period and process (planning and implementation), start of intervals, follow-up appointments, consultation appointments with the physician (Long-term) side effects
	Electronic capture of quality of life and health status or its change
	Timing of therapy change
	Changes in daily life for patients
	Costs and cost coverage of the subsequent therapy
	Palliative medicine, exercise/sports, nutrition, complementary medicine, psycho- and physiotherapy, appropriate information sources
	Contact persons, emergency contact

TTST was not examined as a surrogate endpoint in this study. The table summarizes documentation elements relevant to the operationalization of TTST as an intermediate, care-relevant endpoint and to the assessment of patient relevance

### The Delphi survey

The Delphi drafts were pre-tested for clarity and adjusted.

The first Delphi round targeted physicians, psycho-oncologists, nursing professionals, patient navigators, patients, relatives, advocates, health economists and industry experts. Sample-neutral dropouts included invalid contact details or career changes. Of 2,142 contacted individuals, 264 (12.3%) responded to at least half of the survey and were invited to round two. After 13 sample-neutral dropouts, 251 individuals comprised the second-round target sample; 117 (46.6%) completed the second survey, and 25 partial responses were included. Patients and patient advocates were the largest participant group (see also Table A.4 in the online Appendix).

#### Time to subsequent therapy as a patient-relevant endpoint

**Fig. 1:**
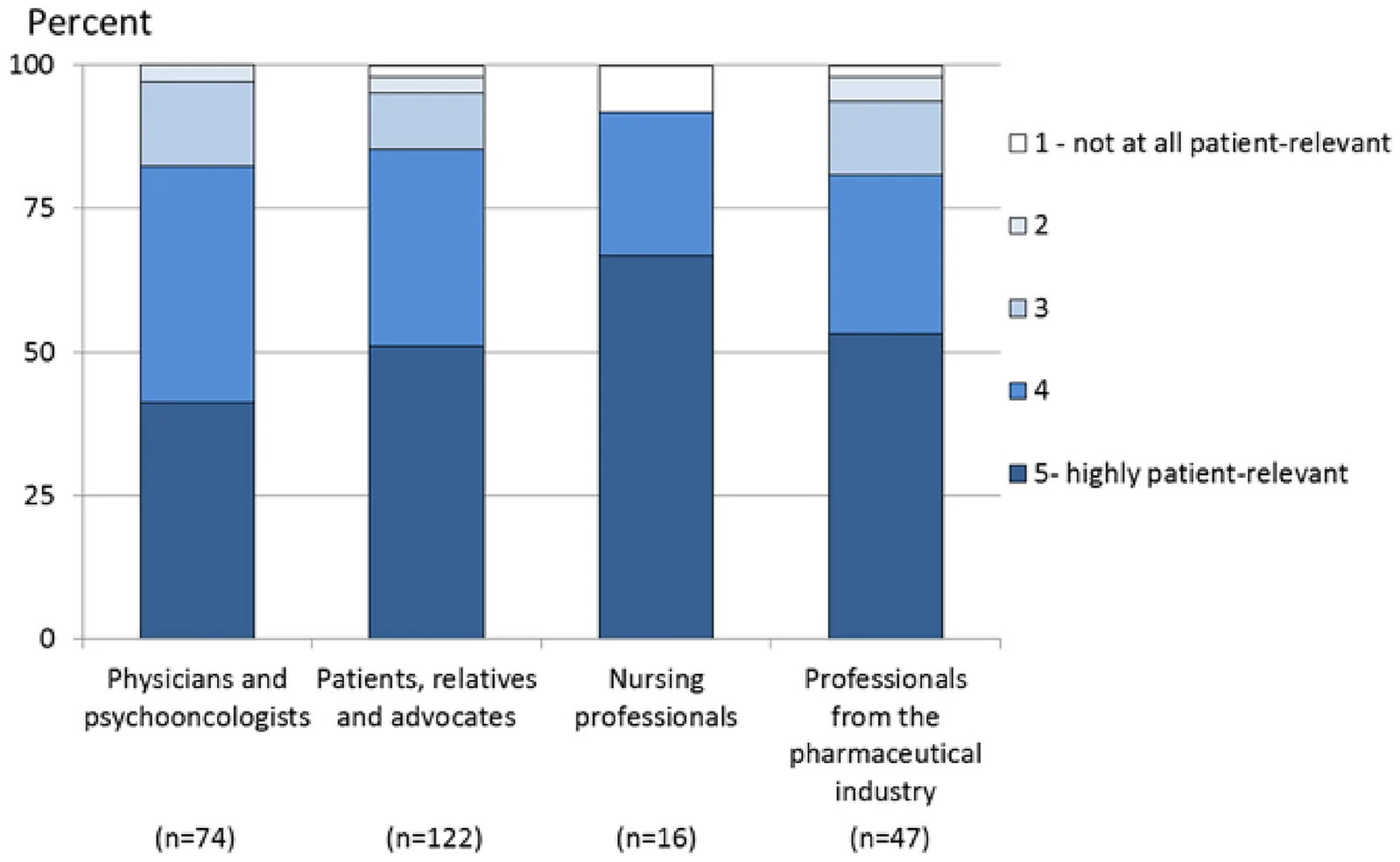
Assessment of patient relevance of the TTST endpoint in the first survey round

In the first Delphi survey, 81% (95% CI 76%, 85%) of all respondents rated TTST as “patient-relevant" or "highly patient-relevant" (for respective groups of interviewees, see Fig. 1). These respondents selected reasons for their decision from a list of possible reasons. Multiple responses were possible. The predominant rationale was that initiation of subsequent therapy can significantly alter patients’ life situation and quality of life of patients, affecting psychological and physical functioning.

Among 19 respondents (6.1%) who denied patient relevance, reasons included the subjective influence of physician decision-making and lack of tangible benefit.

#### Scenarios that are considered to constitute a TTST endpoint

The selection of events reflects the project’s TTST operationalization, and the clinical question addressed by the study. The events listed are those considered relevant for documenting the initiation of subsequent therapy and the context in which treatment decisions are made.

Respondents evaluated 14 possible scenarios for switching to subsequent therapy, with a 50% relevance threshold defining consensus. Nine scenarios met the threshold as TTST events (Table 2). Scenarios such as switching to a similar active substance (within the same ATC class), continuation of the same preparation, temporary treatment breaks, adding further medications, or dose adjustments were generally not classified as TTST events by most respondents. Except for switching within the same ATC class the scenarios were predominantly viewed as patient relevant.

**Table 2:** Scenarios considered potentially relevant for TTST in the 1st Delphi round (n = 254)

	To be included in TTST*	Thereof patient-relevant*
Continuation of treatment with another medicine in case of a relapse	72.3%	77.0%
Switch from monotherapy to combination therapy to improve response rate	69.3%	72.7%
Switch to another treatment with comparable/higher side effect potential	64.8%	72.5%
Switch to another treatment with fewer side effects	64.0%	82.2%
Switch of treatment to a medicine of another substance class	63.6%	61.3%
Switch from combination therapy to monotherapy due to side effects	56.8%	68.7%
Switch to a maintenance therapy upon achieving the treatment goal	53.0%	72.1%
Treatment discontinuation due to side effects without further therapy	52.3%	80.4%
Treatment discontinuation in the face of disease progression without further therapy	51.1%	74.8%
*Treatment switch to a similar active substance (same ATC class)*	*49.2%*	*47.7%*
*Treatment continuation with the same preparation at relapse*	*48.1%*	*61.4%*
*Treatment break due to side effects*	*46.6%*	*80.5%*
*Supportive therapy to reduce side effects*	*38.6%*	*75.5%*
*Dose adjustment of ongoing treatment*	*37.5%*	*66.7%*

^*^Percentages correspond to the result of the first survey round (confirmed in 2nd survey round)

Scenarios listed are those which respondents considered potentially relevant for TTST in the first Delphi round. In the context of a clinical trial, TTST should be defined according to the specific treatment context and the clinical question addressed by the study team. Accordingly, there ís flexibility as to which scenarios are included in a given RCT as TTST-relevant; the short checklist supports this trial-specific operationalization. Italicized scenarios did not reach the predefined 50% threshold. Some italicized scenarios may still be patient-relevant but were outside the TTST definition used for this analysis

**Fig. 2:**
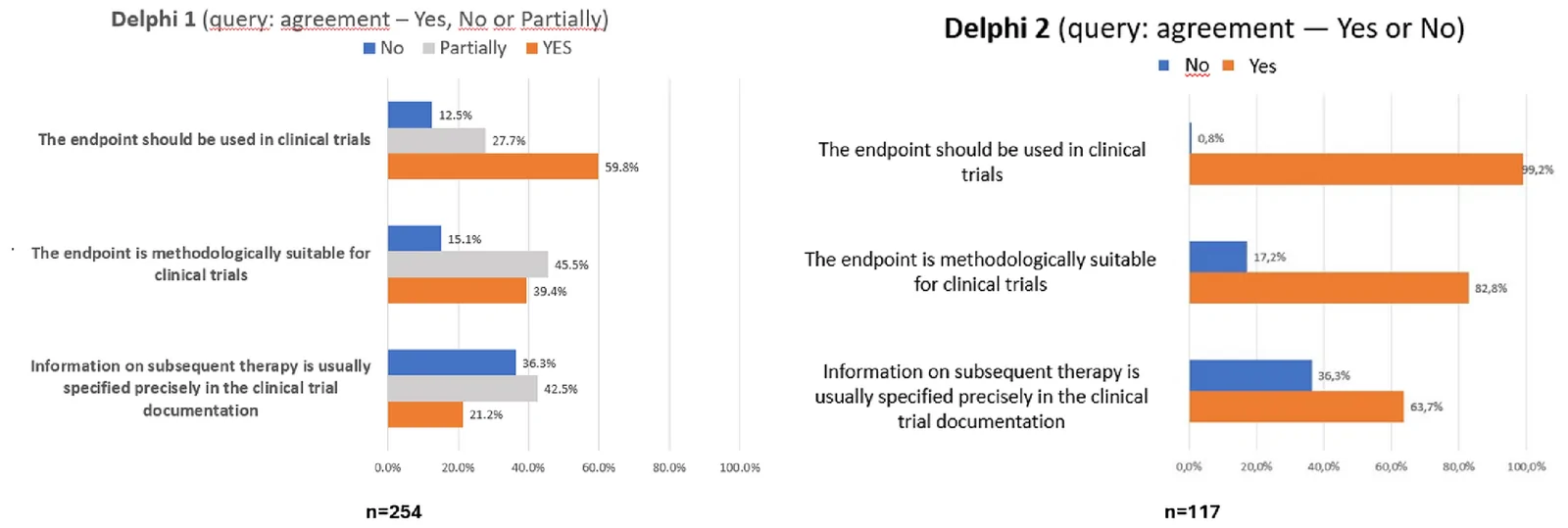
Use and suitability of the TTST endpoint in clinical studies

Five borderline scenarios were re-presented in round two but did not reach the threshold, confirming round-one results. Three further questions assessed overall endpoint suitability (Fig. 2).

Assessments of (a) on necessity of TTST in studies, (b) methodological suitability, and (c) potential redundancy with routine data showed high convergence (Fig. 2). Agreement with statements supporting TTST increased from round one to round two, reaching approximately 64–100% depending on the item.

#### TTST-relevant documentation parameters

To reduce the number of documentation parameters without losing essential information, the Delphi panel rated the relevance and routine availability of each item and advised on format (generic vs. indication-specific; checkbox vs. free-text). Respondents were grouped as: (1) patients / relatives / advocates; (2) physicians / psycho-oncologists; (3) nursing professionals / patient navigators; (4) health economists; (5) industry professionals. Most respondents favored a combined approach: primarily standardized response formats with additional free- text fields for exceptional information (Fig. A.2 in the online Appendix).

### The checklist

The checklist was developed iteratively from SLR, expert interviews, and Delphi results, and benchmarked against de-identified CRFs and G-BA/IQWiG requirements.

Contents were organized along four temporal sections: (1) time before therapy change; (2) time of decision; (3) time of therapy change; and (4) time after implementation of therapy change, enabling clear mapping of required documentation.

A comprehensive master checklist was created and reviewed by vfa Endpoints in Oncology Working Group which provided feedback on applicability and prioritization. The master checklist (Table 3) was revised to distinguish mandatory from optional elements.

**Table 3:** Overview of the versions of the checklist for documenting the TTST endpoint

	Response format^*^	Master checklist	Short version
Study number	Free	X	X
Patient ID	Free	X	X
Date of documentation	Free	X	–
Responsible physician	Free	X	–
Clinic	Free	X	–
Home environment	Fixed	X	–
Age of child/children	Free	X	–
Daily obligations	Fixed/free	X	–
Employment	Fixed/free	X	–
Limitations in daily life	Fixed/free	X	–
Information material & addresses were provided	Date	X	–
Psycho–oncological care	Fixed/Date	X	–
Information material & addresses were provided	Date	X	–
Social medical care	Fixed/Date	X	–
Information material & addresses were provided	Date	X	–
Nutritional medical care	Fixed/Date	X	–
Information material & addresses were provided	Date	X	–
Alternative and complementary medical care	Fixed/Date	X	–
Information material & addresses were provided	Date	X	–
Nursing care	Fixed/Date	X	–
Information material & addresses were provided	Date	X	–
Patient wishes			
Future employment	Fixed/free	X	–
Desire to have children	Fixed	X	–
If desired: Cryopreservation discussed on	Date	X	–
Further wishes of the patient discussed	Fixed/Date	X	–
Expressed wishes of the patient	Free	X	–
Wishes of relatives discussed	Fixed/Date	X	–
Expressed wishes of relatives	Free	X	–
Health status of the patient		X*	X**
Quality of life^1^	Fixed	X*	X**
Health status^2^	Fixed	X*	X**
Pain^3^	Fixed	X*	X**
Mental well-being^4^	Fixed	X*	X**
Reason for therapy change	Fixed/free	X	X
Discussion of all therapy options	Fixed	X	X
Discussion 1st option	Free	X	–
Discussion 2nd option	Free	X	–
Treatment alternatives to the mentioned therapy options	Fixed/free	X	–
Time of patient information regarding therapy change	Date	X	X
Essential contents of the informed consent discussion	Free	X	X
Patient questions were discussed	Date	X	X
Documentation of the specifically discussed questions	Free	X	X
Participation in decision-making/choice of subsequent therapy	Fixed/free	X	–
Responsible party for the final decision/choice of subsequent therapy	Fixed/free	X	–
Procedure for selecting subsequent therapy	Fixed/free	X	–
Description of type and extent of subsequent therapy (including treatment regimen, information on crossover in the comparator arm, etc.)	Free	X	X
Are there toxicities from prior treatments?	Fixed	X	X
Information on unblinding	Free	X	X
Actual start of subsequent therapy	Date	X	X
Time delay in starting subsequent therapy	Fixed/free	X	X
Planned end of subsequent therapy	Date	X	X
Description of type and extent of follow-up after start of subsequent therapy	Free	X	X
Termination of subsequent therapy	Fixed/free	X	X

Response format* → fixed: pre-determined response categories including checkboxes; free: free text entries; Date: date entries

^1+2^ = European organization for research and treatment of cancer—core quality of life questionnaire (EORTC-QLQ-C30)

^3^ = Veterans rand 36 item health survey (VR-36) (Kazis et al. 2006) or SF-36

^4^ = World mental health composite international diagnostic interview (WMH-CIDI)

^*^ Documentation of the patient’s health status at each time point

^**^ Documentation of the patient’s health status at the time of decision-making/therapy change and follow-up

Finally, a short checklist on core investigator elements for regulatory or benefit assessment studies was produced, yielding two complementary instruments: a full master checklist for complex needs, e.g., in health services research (Table 3), and a reduced short version for clinical trials with pre-specified TTST endpoints (Table 4).

**Table 4:**
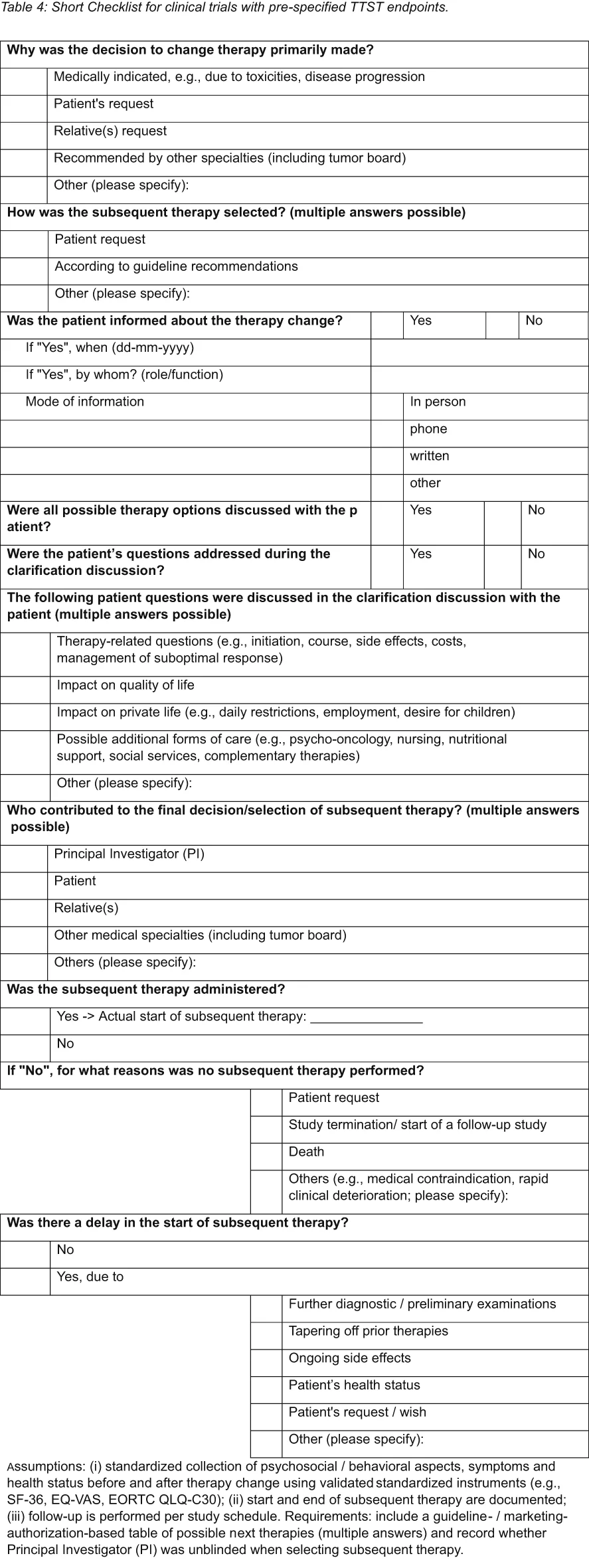
Short checklist for clinical trials with pre-specified TTST endpoints

## Discussion

Time To Subsequent Therapy (TTST) is the time from index treatment to the initiation of subsequent therapy (Agapow et al. 2023). In this context, it is also worth briefly addressing the Estimand Framework. This framework defines different strategies for handling “intercurrent events post randomization.” Within the estimand framework (https://www.ema.europa.eu/en/documents/scientific-guideline/appendix-1-guideline-evaluation-anticancer-medicinal-products-man-methodological-consideration-using-progression-free-survival-or-disease-free-survival-confirmatory-trials_en.pdf (ICH 2019)), the EMA recommendation to document the end of subsequent therapy line applies to a specific clinical question. Also in this context, subsequent therapy and related post-treatment events, such as progression, discontinuation, or death, are treated as intercurrent events whose handling determines how TTST is interpreted; this is also the context in which patient relevance becomes particularly important. Therefore, TTST should be interpreted in relation to the specific clinical question and the handling of subsequent therapy and related intercurrent events. The results of the Delphi survey suggest that the classification of a scenario as TTST-relevant depends on whether the event represents a failure of the current treatment strategy, a planned step in a pre-specified treatment sequence, or a minor modification within ongoing treatment.

Accordingly, the scenarios listed in Table 2 reflect how TTST was operationalized for the respective clinical context and were selected to support consistent documentation of relevant therapy-change situations. While the Delphi survey yielded descriptive treatment patterns only, the variation across scenarios indicated that some events may be better understood as minor treatment modifications within ongoing management, whereas others represent an actual transition to subsequent therapy. Scenarios with lower agreement, such as temporary treatment breaks or dose adjustments, may be interpreted as treatment-policy components in some contexts, whereas planned switching scenarios (e.g., switching to maintenance therapy) require prospective specification. Conversely, switches due to relapse, toxicity, or progression-related events may be more naturally compatible with TTST-defining scenarios. For scenarios that may be treatment-strategy-related, either planned sequencing or unplanned treatment failure, the protocol should specify in advance whether they count as TTST events. This is especially relevant for switches to maintenance therapy, switches within treatment class, and transitions from monotherapy to combination therapy or combination therapy to monotherapy.

As with other time-to-event endpoints influenced by subsequent therapy, the interpretation of TTST depends on the underlying clinical question and on how events occurring after the index therapy are handled. In some settings, subsequent therapy may be considered an intercurrent event; in others, particularly sequencing trials, it may form part of the treatment strategy under investigation. This distinction is important when interpreting TTST and should be made explicit in the respective study context. Unlike PFS2, as recently discussed by FDA (Fiero et al. 2026), TTST in the present project was not intended to serve as a surrogate endpoint, but rather to improve the documentation needed to assess TTST consistently and transparently as a care-relevant intermediate endpoint. The patient relevance of TTST arises not only from the timing of a new therapy, but from the consequences associated with the treatment change, including loss of disease control, treatment burden, functional decline, psychological distress, and transition in care goals.

This work contributes to improved TTST documentation by standardizing data collection, thereby enhancing data quality, comparability across studies, and the assessment of patient-relevant intermediate outcomes.

The collection of endpoints using standardized, validated instruments is central to clinical research and underpins efficacy, safety and health-technology benefit assessments. In German AMNOG benefit assessments, patient relevance of endpoints is a key consideration (Dabisch et al. 2014). European HTA Heads and the European Medicines Agency (EMA) have pointed out that ‘non-primary’ endpoints should be given more consideration in the development of clinical studies (https://www.ema.europa.eu/en/documents/other/joint-htab-regulatory-perspectives-understanding-evidence-challenges-managing-uncertainties-exploring-potential-solutions_en.pdf). Company AMNOG dossiers in G-BA benefit assessment procedures reported TTST results in a total of 73 procedures (AMNOG-Monitor 2025).

TTST may have different meanings across oncology indications because treatment standards, disease trajectories, and reasons for changing therapy vary by tumor type. Accordingly, the checklist was designed as a flexible framework that captures a common core of TTST-relevant information while allowing indication-specific adaptation where necessary.

The endpoint TTST has gained traction in clinical and health services research. In oncology, due to the availability of new therapy options, the chance of extending overall survival beyond the actual study duration has sometimes increased dramatically. On the other hand, at more advanced stages of disease, no further causally effective oncological therapies may be available, so that the tolerability and efficacy of later therapy lines become essential. Similar dynamics occur in other chronic diseases (e.g., rheumatic or respiratory conditions). Therefore, intermediate endpoints that capture treatment effects are sought. TTST is of particular interest because it reflects therapy durability, symptom control, and patient-level factors such as compliance, adherence and treatment-related behavior (Campbell et al. 2020). Given that regulatory authorities consider TTST relevant (Olaparib 2015; https://www.ema.europa.eu/en/documents/scientific-guideline/appendix-1-guideline-evaluation-anticancer-medicinal-products-man-methodological-consideration-using-progression-free-survival-or-disease-free-survival-confirmatory-trials_en.pdf), evidence of its patient-relevance should be assessed in the RCTs that inform such evaluations.

In health services research, TTST has been used across various of oncologic entities, underlining its practical relevance (Rivera et al. 2022). The EMA recommends recording the end of the subsequent therapy line when overall survival and progression-free survival under subsequent therapy cannot be reliably determined, defining the event as end or discontinuation of the subsequent therapy, progression of the cancer disease under subsequent therapy, or death from any cause, whichever occurs first (https://www.ema.europa.eu/en/documents/scientific-guideline/appendix-1-guideline-evaluation-anticancer-medicinal-products-man-methodological-consideration-using-progression-free-survival-or-disease-free-survival-confirmatory-trials_en.pdf).

The project aimed to assess the patient relevance of the intermediate endpoint TTST and to develop a checklist to standardize data collection and to make the information needed to evaluate patient relevance transparent and usable.

In the reviewed period, G-BA/IQWiG did not consider TTST patient-relevant in company-submitted clinical trials. Major reasons included lack of post-therapy follow-up of relevant variables, non-pre-specified definition of TTST, initiation of subsequent therapy based solely on imaging, inadequate documentation of treatment decisions and context, and/or investigator unblinding after original treatment. These shortcomings motivated the development of documentation guidance because the reasons given by G-BA/IQWiG for not considering TTST as patient-relevant were closely tied to the specific way TTST had been implemented in individual submissions, particularly missing follow-up of relevant patient-centered outcomes, retrospective endpoint definitions, limited documentation of treatment decisions, and concerns about unblinding. These concerns do not question TTST as such but rather highlight that its interpretability depends on the clinical question, the endpoint definition, and the associated estimand. The present checklist was developed to address these documentation gaps and to support a more transparent and patient-centered operationalization of TTST. These concerns highlight that TTST classifications depend on the estimand and the handling of intercurrent events, which should be prospectively defined.

Qualitative sub-studies (SLR, document analysis, expert interviews) identified multiple determinants of TTST. Patient-level factors include symptoms under previous therapy, toxicity, functional limitations, quality of life and mental state changes, psychosocial and behavioral aspects, age, experience and disease knowledge. Care- and clinician-related factors include treating-physician’s expertise, available therapy options, type of therapy, monitoring intervals, and whether progression is detected with or without symptoms (e.g., by imaging). The patient’s social environment also influences decisions about subsequent therapy.

Participation in the first Delphi round was modest (264/2142 invited; 12.3%), which may reflect the concurrent Covid-19 pandemic. Participation improved substantially in round two, with 117/251 (46.6%) respondents completing the survey.

The two-stage Delphi quantitatively supported patient relevance of TTST and helped structure potential documentation items. Based on this work, two checklists were developed:

a) A comprehensive master checklist for health service research and routine care studies, adaptable and covering broad documentation need; and.

b) A short checklist for clinical trials with pre-specified TTST endpoints, focusing on essential items to justify patient relevance.

Only 6.1% of Delphi respondents considered TTST not patient relevant. Critical objections included relevance mainly at the curative-to-palliative-transition, endpoint neutrality, subjective physician decision, and the primacy of quality of life and overall survival. Our findings temper these concerns: TTST is particularly pertinent with modern therapies in palliative situations, the checklists promote participatory and transparent decision-making, and quality of life and overall survival remain captured within recommended documentation.

Criticism from HTA in prior benefit assessments on TTST focused on insufficient and unsystematic documentation of therapy changes. This is mainly in Germany because in other jurisdictions PFS data are more routinely considered and drive discussions (Dabisch et al. 2014). The developed checklists aim to address these deficiencies and should be empirically tested for validity, feasibility and usefulness in future studies, for example via prospective integration into ongoing clinical studies or dedicated methodological studies.

The project was designed primarily for the German HTA context, but the checklist elements related to therapy-change documentation may also be informative for broader international settings, especially if overall survival data are not mature at time of market authorization.

## Limitation

Limitations include spatial and temporal restrictions of the literature and document analysis and potential selection bias in the surveys, especially during the COVID-19 pandemic. Newer, post-COVID approaches to patient involvement (e.g., patient-informed search term development) were not performed. However, patient interviews were relatively open-structured, allowing participants to express perspectives, and the sample size and diversity support robust conclusions. Methodological triangulation across approaches yielded consistent results: TTST can be considered patient-relevant if standardized, pre-defined documentation is implemented.

## Conclusion

To date, TTST has often been reported without documentation of patient involvement in decision-making. Now a new TTST checklist is available to improve capture of patient-relevant aspects. The master checklist (for health services research) and the short checklist (for registration trials) provide standardized tools applicable in oncologic studies and beyond.

## Supplementary Information

Below is the link to the electronic supplementary material.

Supplementary file1 (DOCX 671 kb)

## Data Availability

The datasets used and/or analysed during the current study are available from the corresponding author on reasonable request.
