# Supplementary material for: Time to subsequent therapy (TTST) as an endpoint in clinical studies: development of standardized documentation of subsequent therapy through systematic literature review, expert interviews, and Delphi survey
Source: J Cancer Res Clin Oncol. 2026 Sep 2;152(9):179. doi: 10.1007/s00432-026-06583-w (PMC13578166; doi:10.1007/s00432-026-06583-w)
Supplement: Supplementary file 1 [file 432_2026_6583_MOESM1_ESM.docx]

**Appendix: Time to Subsequent Therapy (TTST) as an Endpoint in Clinical Studies: Development of Standardized Documentation of Subsequent Therapy Through Systematic Literature Review, Expert Interviews, and Delphi Survey**

# Appendix

# **Table A.1: Keywords of the literature search**

| **Primary search terms** | **Combination terms*** |
| --- | --- |
| TTST | oncological studies/ clinical trials/ oncological therapy/ cancer clinical trials |
| Time to next therapy | Endpoints/ Endpoints (primary)/ surrogate endpoints |
| Time to start of therapy | Patient involvement/ patient participation |
| Therapy switch | Patient preferences/ Patient preference |
| Therapy adjustment | Additional benefit/ additional benefit |
| time to subsequent therapy | Patient relevance/ patient-relevant |
| time to progression | health-related quality of life/ health related quality of life |
| time to treatment failure | Overall survival |
| time to next treatment | progression-free survival |
| time to first subsequent therapy | disease-free survival |
| time to second subsequent therapy | event-free survival |
|  | Therapy change |
|  | therapy adjustment |
|  | assessment of outcome measures |
|  | patient-reported outcome measures |
|  | patient-reported experience measure |
|  | IQWiG/ GBA/ EMA /FDA |

* The primary search terms were each combined with all combination terms and searched in the literature search

**Table A.2: Summary of results of the literature search**

# **Table A.3: Results of the search for benefit assessments for the five study-relevant indications**

| **Active substance** | **TTST-related endpoints** | **Reasons for exclusion of endpoints** | **Reference [Accessed 26.10.2025]** |
| --- | --- | --- | --- |
| ***Breast cancer*** | | | |
| Abemaciclib  (2019) | Time from randomization to end of treatment of the next line of therapy or to start of treatment of a further line of therapy/ Time from randomization to start of subsequent chemotherapy or death | Post-hoc definition, missing follow-up, follow-up therapy after progression based on imaging procedures, circumstances of treatment decision not collected | IQWiG Report 30.01.2019 <https://www.g-ba.de/downloads/92-975-2708/2018-11-01_Nutzenbewertung-IQWiG_Abemaciclib-D-401.pdf> |
| Abemaciclib  (2020) | Time to end of next therapy line, time to first subsequent chemotherapy, time to first subsequent intravenous chemotherapy | Lack of detailed information on  post-progression therapies, furthermore essential information about the  circumstances of the treatment decision for or against chemotherapy is not described by the  pharmaceutical company, significant uncertainties regarding the validity of the results for this endpoint therefore no additional benefit derivable | 15.03.2020 [Benefit assessment procedure for the active substance Abemaciclib (Re-assessment after deadline expiry: Breast cancer, HR+, HER2-, combination with Fulvestrant) - Federal Joint Committee](https://www.g-ba.de/bewertungsverfahren/nutzenbewertung/535/) |
| Abemaciclib  (2021) | Time to 1st subsequent chemotherapy | No additional benefit derivable due to significant uncertainties regarding the validity of the endpoint results | 01.12.2021 [Benefit assessment procedure for the active substance Abemaciclib (Re-assessment after deadline expiry: Breast cancer, HR+, HER2-, combination with Fulvestrant) - Federal Joint Committee](https://www.g-ba.de/bewertungsverfahren/nutzenbewertung/767/#dossier) |
| Abemaciclib  (2023) | Time to first subsequent chemotherapy | Patient relevance questionable, significant uncertainties regarding the validity of the endpoint results | 01.01.2023 <https://www.g-ba.de/bewertungsverfahren/nutzenbewertung/911/> |
| Palbociclib  (2019) | Time from randomization to start of subsequent chemotherapy or death | Post-hoc definition, missing follow-up (morbidity, health-related quality of life, side effects), circumstances of treatment decision not collected | IQWiG Report 23.02.2017 <https://www.g-ba.de/downloads/92-975-1746/2017-03-01_Nutzenbewertung-IQWiG_Palbociclib-D-264.pdf> Transcript GBA 11.02.2019 <https://www.g-ba.de/downloads/91-1031-394/2019-02-11_Wortprotokoll_Palbociclib_D-395.pdf> , Supporting reasons GBA 22.03.2019 <https://www.g-ba.de/downloads/40-268-5642/2019-03-22_AM-RL-XII_Palbociclib_D-395_TrG.pdf> |

| Palbociclib  (2022) | Time to first subsequent chemotherapy | Minor additional benefit is described, no mention of endpoint in the resolution | 01.01.2023 <https://www.g-ba.de/bewertungsverfahren/nutzenbewertung/845/> |
| --- | --- | --- | --- |
| Ribociclib  (2019) | Time to first subsequent chemotherapy | Missing follow-up, possible delay of subsequent chemotherapy due to side effects of the drug | IQWiG Report 13.12.2017 <https://www.g-ba.de/downloads/92-975-2079/2017-12-15_Nutzenbewertung-IQWiG_Ribociclib-D-307.pdf> |
| Ribociclib  (2020) | Time to first subsequent chemotherapy | Data collection beyond discontinuation of study medication necessary but not documented | 01.03.2020 [Benefit assessment procedure for the active substance Ribociclib (Re-assessment after deadline expiry: Breast cancer, HR+, HER2-, combination with an aromatase inhibitor) - Federal Joint Committee](https://www.g-ba.de/bewertungsverfahren/nutzenbewertung/526/) |
| Olaparib  (2019) | Time to subsequent therapy (summarized from time to 1st and 2nd subsequent therapy, time to 1st and 2nd subsequent chemotherapy) | not the start of a subsequent therapy is relevant as such, but the question of which patient-relevant consequences (e.g., side effects) result from the use of the subsequent therapy, this was not documented | 15.07.2019 [Benefit assessment procedure for the active substance Olaparib (new indication: Breast cancer, BRCA1/2 mutations, HER2-) - Federal Joint Committee](https://www.g-ba.de/bewertungsverfahren/nutzenbewertung/463/) |
| Atezolizumab  (2019) | Time to next Treatment | Rejection, as only the results were mentioned and no additional information on the endpoint is available | 01.10.2019 [Benefit assessment procedure for the active substance Atezolizumab (new indication: Breast cancer, triple-negative, PD-L1 expression ≥1%) - Federal Joint Committee](https://www.g-ba.de/bewertungsverfahren/nutzenbewertung/493/) |
| Neratinib  (2019) | Time to subsequent therapy | To show a possible advantage of extended adjuvant  therapy on patient-relevant endpoints, data collection  beyond discontinuation of study medication would be necessary for these endpoints. | 01.12.2019 [Benefit assessment procedure for the active substance Neratinib (Breast cancer, HR+, HER2+, adjuvant therapy) - Federal Joint Committee](https://www.g-ba.de/bewertungsverfahren/nutzenbewertung/510/#nutzenbewertung) |
| Talazoparib  (2020) | Time to start of first subsequent therapy | *No statement on endpoint* | 01.06.2020 [Benefit assessment procedure for the active substance Talazoparib (Breast cancer, BRCA1/2 mutation, HER2-) - Federal Joint Committee](https://www.g-ba.de/bewertungsverfahren/nutzenbewertung/554/) |
| Alpelisib  (2020) | Time to 1st subsequent chemotherapy | *No statement on endpoint* | 01.09.2020 [Benefit assessment procedure for the active substance Alpelisib (Breast cancer with PIK3CA mutation, pretreated patients, combination with Fulvestrant) - Federal Joint Committee](https://www.g-ba.de/bewertungsverfahren/nutzenbewertung/581/) |

| Pembrolizumab  (2021) | Time to 1st subsequent therapy or death | Endpoint not patient-relevant | 15.11.2021 [Benefit assessment procedure for the active substance Pembrolizumab (New indication: Breast cancer, triple-negative, PD-L1 expression ≥ 10 (CPS), combination with chemotherapy) - Federal Joint Committee](https://www.g-ba.de/bewertungsverfahren/nutzenbewertung/757/) |
| --- | --- | --- | --- |
| Elacestrant  (2023) | Time to chemotherapy | *No statement on endpoint* | 01.11.2023 <https://www.g-ba.de/bewertungsverfahren/nutzenbewertung/1007/> |
| ***Multiple Myeloma*** | | | |
| Pomalidomide  (2019) | Time to next Treatment | Advantages of the endpoint are recognized, but further data are missing | 15.06.2019 [Benefit assessment procedure for the active substance Pomalidomide (new indication: Multiple myeloma, at least 1 prior therapy, combination with Bortezomib and Dexamethasone) - Federal Joint Committee](https://www.g-ba.de/bewertungsverfahren/nutzenbewertung/467/) |
| Daratumumab  (2020) | Time from randomization to start of subsequent myeloma therapy or to death | Initiation of subsequent therapy after progression based on imaging procedures | IQWiG Report 20.12.2018 <https://www.g-ba.de/downloads/92-975-2624/2018-10-01_Nutzenbewertung-IQWiG_Daratumumab-D-403.pdf> |
| Daratumumab  (2022) | Subsequent therapy after platinum-based therapy | *No statement on endpoint* | 01.04.2022 [https://www.g-ba.de/bewertungsverfahren/nutzenbewertung/809/](https://www.g-ba.de/bewertungsverfahren/nutzenbewertung/809/%20) |
| Carfilzomib  (2021) | Time to start of subsequent therapy | Missing follow-up (symptoms, health-related quality of life, adverse events) | IQWiG Report 13.11.2017 <https://www.g-ba.de/downloads/92-975-2031/2017-08-15_Nutzenbewertung-IQWiG_Carfilzomib-D-302.pdf> |
| Ixazomib  (2021) | Time to first antineoplastic subsequent therapy | can be interpreted as a surrogate for the duration of treatment efficacy in disease and symptom control | 01.11.2021 [Benefit assessment procedure for the active substance Ixazomib (Re-assessment after deadline expiry: Multiple myeloma, at least 1 prior therapy, combination with lenalidomide and dexamethasone) - Federal Joint Committee](https://www.g-ba.de/bewertungsverfahren/nutzenbewertung/751/) |
| Idecabtagene vicleucel  (2022) | Time to subsequent myeloma therapy | Endpoint not operationalized, no patient relevance demonstrated | G-BA Assessment 2022  <https://www.g-ba.de/bewertungsverfahren/nutzenbewertung/781/> |
| Melphalan flufenamide  (2022) | Time until new treatment becomes necessary/Time to subsequent therapy | *No statement on endpoint* | 01.10.2022 [https://www.g-ba.de/bewertungsverfahren/nutzenbewertung/883/](https://www.g-ba.de/bewertungsverfahren/nutzenbewertung/883/%20) |

| Selinexor  (2022) | Time to next treatment | No statement on the endpoint IQWiG indicates that therapy changes occurred too early; thus the interpretability of the subgroup results is not given | 01.10.2022 <https://www.g-ba.de/bewertungsverfahren/nutzenbewertung/886/> |
| --- | --- | --- | --- |
| Ciltacabtagene autoleucel  (2023) | Time to subsequent myeloma therapy | Consideration in benefit assessment: No due to unclear patient relevance, operationalization of the endpoint limited comprehensible | 15.02.2023 <https://www.g-ba.de/bewertungsverfahren/nutzenbewertung/924/> |
| Teclistamab  (2023) | Time to subsequent myeloma therapy | *No statement on endpoint* | 01.09.2023 <https://www.g-ba.de/bewertungsverfahren/nutzenbewertung/989/> |
| Talquetamab  (2023) | Time to subsequent myeloma therapy | *No statement on endpoint* | 15.09.2023 <https://www.g-ba.de/bewertungsverfahren/nutzenbewertung/997/> |
| Elranatamab  (2024) | Time to subsequent therapy | *No statement on endpoint* | 15.04.2024 [https://www.g-ba.de/bewertungsverfahren/nutzenbewertung/1051/](https://www.g-ba.de/bewertungsverfahren/nutzenbewertung/1051/%20) |
| Idecabtagene vicleucel  (2024) | Time to subsequent myeloma therapy | *No statement on endpoint* | 01.04.2024 <https://www.g-ba.de/bewertungsverfahren/nutzenbewertung/1068/> |
| ***Non-small cell lung carcinoma*** | | | |
| Alectinib  (2019) | Time to subsequent therapy (drug therapy or radiotherapy) | Missing follow-up, initiation of subsequent therapy after progression based on imaging procedures | IQWiG Report 28.03.2018 <https://www.g-ba.de/downloads/92-975-2247/2018-01-01_Nutzenbewertung-IQWiG_Alectinib-D-326.pdf> , oral hearing GBA 08.05.2018 <https://www.g-ba.de/downloads/91-1031-339/2018_05_08_Wortprotokoll_Alectinib-D-326.pdf> , Summary documentation GBA 21.06.2018 <https://www.g-ba.de/downloads/40-268-5215/2018-06-21_AM-RL-XII_Alectinib_D-326_ZD.pdf> |
| Durvalumab  (2019) | Time from randomization to start of first subsequent therapy after treatment discontinuation or to death | Missing follow-up (symptoms, health-related quality of life, adverse events), initiation of subsequent therapy after progression based on imaging procedures | IQWiG Report 01.10.2019 <https://www.g-ba.de/downloads/92-975-2690/2018-10-15_Nutzenbewertung-IQWiG_Durvalumab-D-402.pdf> |
| Osimertinib  (2019) | Time from randomization to 1st or 2nd subsequent therapy after discontinuation of randomized treatment or to death | Missing follow-up, initiation of subsequent therapy after progression based on imaging procedures | IQWiG Report 10.11.2018 <https://www.g-ba.de/downloads/92-975-2507/2018-07-15_Nutzenbewertung-IQWiG_Osimertinib-D-369.pdf> |
| Osimertinib  (2021) | Time to first subsequent therapy | *No statement on endpoint* | 01.07.2021 [Benefit assessment procedure for the active substance Osimertinib (New indication: Non-small cell lung cancer, EGFR mutations, adjuvant therapy) - Federal Joint Committee](https://www.g-ba.de/bewertungsverfahren/nutzenbewertung/713/) |

| Osimertinib  (2024) | Time to first subsequent therapy | *No statement on endpoint* | 01.07.2024 <https://www.g-ba.de/bewertungsverfahren/nutzenbewertung/1096/> |
| --- | --- | --- | --- |
| Osimertinib  (2024) | Time to first subsequent therapy; Time to second subsequent therapy | *No statement on endpoint* | 01.08.2024 <https://www.g-ba.de/bewertungsverfahren/nutzenbewertung/1107/> |
| Pembrolizumab  (2019) | Time to therapy change | *No statement on endpoint* | Dossier GBA 02.09.2017 <https://www.g-ba.de/downloads/92-975-1803/2017-02-09_Modul4A_Pembrolizumab.pdf> |
| Pembrolizumab  (2024) | Time to first subsequent therapy; Time to first subsequent surgery | *No statement on endpoint* | 01.05.2024 <https://www.g-ba.de/bewertungsverfahren/nutzenbewertung/1026/> |
| Pembrolizumab  (2024) | Time to first subsequent therapy; Time to first subsequent surgery | Endpoint shows benefit but result cannot be included in the benefit assessment | 01.05.2024 <https://www.g-ba.de/bewertungsverfahren/nutzenbewertung/1082/> |
| Ramucirumab  (2020) | Time to first subsequent systemic therapy or death | *No statement on endpoint* | 15.02.2020 [Benefit assessment procedure for the active substance Ramucirumab (new indication: non-small cell lung cancer, EGFR mutation, first line) - Federal Joint Committee](https://www.g-ba.de/bewertungsverfahren/nutzenbewertung/523/) |
| Amivantamab  (2022) | Time to start of next therapy | *No statement on endpoint* | 15.01.2022 <https://www.g-ba.de/bewertungsverfahren/nutzenbewertung/783/> |
| Sotorasib  (2022) | Time to next line of therapy | *No statement on endpoint* | 15.02.2022 <https://www.g-ba.de/bewertungsverfahren/nutzenbewertung/799/> |
| ***Prostate cancer*** | | | |
| Abiraterone acetate  (2019) | Time to subsequent therapy/ Time to life-prolonging subsequent therapy | Circumstances of treatment decision not collected, unblinding of study physician after end of therapy | IQWiG Report 13.03.2018 , T <https://www.g-ba.de/downloads/92-975-2220/2017-12-15_Nutzenbewertung-IQWiG_Abirateronacetat-D-337.pdf> Justification GBA 06.07.2018 <https://www.g-ba.de/downloads/40-268-5049/2018-06-07_AM-RL-XII_Abirateronacetat-nAWG_D-337_TrG.pdf> , Summary documentation GBA 06. and 07.07.2018 <https://www.g-ba.de/downloads/40-268-5270/2018-06-07_AM-RL-XII_Abirateronacetat-nAWG_D-337_ZD.pdf> |

| Enzalutamide  (2019) | Time to initiation of new antineoplastic therapy or cytotoxic chemotherapy | Missing follow-up, circumstances of treatment decision not collected | IQWiG Report 26.02.2019 <https://www.g-ba.de/downloads/92-975-2785/2018-12-01_Nutzenbewertung-IQWiG_Enzalutamid_D-411.pdf> and Addendum 25.04.2019 <https://www.g-ba.de/downloads/92-975-2969/2019-05-16_Addendum-IQWiG_Enzalutamid-nAWG_D-411.pdf> , Justification GBA 16.05.2019 <https://www.g-ba.de/downloads/40-268-5744/2019-05-16_AM-RL-XII_Enzalutamid_D-411_TrG.pdf> , Transcript GBA 08.04.2019 <https://www.g-ba.de/downloads/91-1031-417/2019_04_08_Wortprotokoll_Enzalutamid_D-411.pdf> |
| --- | --- | --- | --- |
| Enzalutamide  (2020) | Time to initiation of new antineoplastic therapy or cytotoxic chemotherapy | Missing data collection beyond the entire period | 15.05.2020 [Benefit assessment procedure for the active substance enzalutamide (Re-assessment after deadline expiry: Prostate carcinoma, non-metastatic, high-risk) - Federal Joint Committee](https://www.g-ba.de/bewertungsverfahren/nutzenbewertung/551/) |
| Enzalutamide  (2021) | Time to initiation of new antineoplastic therapy or cytotoxic chemotherapy | Missing data collection beyond the entire period | 01.06.2021 [Benefit assessment procedure for the active substance enzalutamide (New therapeutic indication: Prostate carcinoma, metastatic, hormone-sensitive, combination with androgen deprivation therapy) - Federal Joint Committee](https://www.g-ba.de/bewertungsverfahren/nutzenbewertung/696/) |
| Olaparib  (2023) | Time to first chemotherapy, Time to first radiotherapy (Time to first subsequent Therapy) | Endpoint is already represented in other endpoints | 15.01.2023 <https://www.g-ba.de/bewertungsverfahren/nutzenbewertung/917/> |
| ***Melanoma*** | | | |
| Pembrolizumab  (2022) | Time to first subsequent therapy or death | *No statement on endpoint* | 01.08.2022 <https://www.g-ba.de/bewertungsverfahren/nutzenbewertung/855/> |
| Pembrolizumab  (2022) | Time to first subsequent therapy or death | *No statement on endpoint* | 01.08.2022 <https://www.g-ba.de/bewertungsverfahren/nutzenbewertung/856/> |

# **Table A.4: Willingness to participate by survey groups**

| **Survey group** | **1st Delphi round** | | | **2nd Delphi round** | | |
| --- | --- | --- | --- | --- | --- | --- |
|  | Invited to participate | participated | *Participation rate in %* | Invited to participate | participated | *Participation rate in %* |
| Patients, relatives, and patient advocates | 824 | 122 | *14.8* | 114 | 60 | *52.6* |
| Physicians and Psycho-oncologists | 982 | 74 | *7.5* | 70 | 24 | *34.3* |
| Nursing professionals and patient navigators | 119 | 16 | *13.4* | 13 | 7 | *53.8* |
| Health economists | 16 | 5 | *31.3* | 6 | 6 | *100.0* |
| Professionals from the pharmaceutical industry | 201 | 47 | *23.4* | 48 | 20 | *41.7* |
| Total | 2142 | 264 | *12.3* | 251 | 117 | *46.6* |

**Figure A.1 Recommendation for inclusion in a TTST documentation tool and recording in standard study documents – Delphi Panel.** The Y-axis shows the frequency with which a documentation point was recommended for inclusion in TTST documentation. The X-axis shows whether the corresponding documentation point is already recorded in standard study documents according to the respondents. The upper left area contains documentation points that are considered highly relevant and that, according to the respondents, are already recorded in standard study documents. The middle area contains documentation points that were classified as TTST-relevant by more than half of the respondents but are rather not already recorded in standard study documentation. Documentation points in the lower right area are currently rather missing from existing documentation.
